# Correlation Between Sialidase NEU1 mRNA Expression Changes in Autism Spectrum Disorder

**DOI:** 10.3389/fpsyt.2022.870374

**Published:** 2022-06-09

**Authors:** Haiqing Zhang, Yuhang Gu, Wenxiang He, Fengyi Kuo, Yiran Zhang, Duan Wang, Li He, Ying Yang, Hepeng Wang, Yanni Chen

**Affiliations:** ^1^Department of Child Healthcare, Xi'an Children's Hospital, Xi'an, China; ^2^Department of Pediatrics, Ankang Maternal and Child Health Hospital, Ankang, China; ^3^LIH Healthcare, Beijing, China; ^4^Shaanxi Institute for Pediatric Diseases, Xi'an Children's Hospital, Xi'an, China; ^5^Department of Pediatrics, Shaanxi University of Chinese Medicine, Xianyang, China

**Keywords:** autism spectrum disorder, sialidase NEU1, gene expression, Language and Communication, ADOS-2

## Abstract

Abnormal alterations in enzymes functioned in sialic acid modifications may be associated with ASD. In order to study the differences in peripheral blood sialidase (neuraminidase 1; NEU1) mRNA expression between autism spectrum disorder (ASD) children and healthy control, and to examine the correlation between NEU1 mRNA expression and the main behavioral phenotypes in children with ASD, we performed RT-qPCR to measure NEU1 mRNA expression in peripheral blood of 42 children with ASD and 42 healthy controls. In addition, we used the Autism Diagnostic Observation Schedule, Second Edition (ADOS-2) to measure and evaluate the behavioral phenotypes of children with ASD. Our results showed that NEU1 mRNA in the ASD group was significantly higher than in the control group (*P* < 0.0001). In addition, the ADOS-2 diagnostic scores of 42 children with ASD were correlated with their NEU1 mRNA expression results (*R* = 0.344, *P* = 0.0257). Moreover, in general, NEU1 mRNA expression was also positively correlated with the Social Affect (SA) of ADOS-2 (*R* = 0.3598, *P* = 0.0193) but not with the Restricted and Repetitive Behavior (RRB) (*R* = 0.15, *P* = 0.3432). Our results indicated that sialidase NEU1 mRNA was significantly increased in children with ASD, and its expression was correlated with the SA of children with ASD, which suggested that sialidase NEU1 may affect the SA of ASD. Our data highlighted the potential of NEU1 expression change may play an important role in ASD disease and lay the foundation for further studies on the relationship between NEU1 and ASD.

## Introduction

Autism spectrum disorder (ASD) is a complicated neurodevelopmental disorder with clinical characterizations of social and communication deficits, as well as repetitive and stereotyped behaviors (1). According to the Centers for Disease Control and Prevention (CDC), the latest prevalence rate of ASD in 8-year-old children in the United States is 1/44 (~2.27%), which has become a social problem (2). Despite the high prevalence rate, the pathophysiology of ASD remains elusive. Previously, multiple studies suggested abnormal glycosylation as an emerging research direction for the etiology of ASD (3–5). Sialylation belongs to one of the types of glycosylation, it functions by adding sialic acid to growing glycan chains on glycoproteins and glycolipids (6, 7). In our previous study, we used lectin microarrays and lectin-magnetic particle conjugate-assisted liquid chromatography with tandem mass spectrometry (LC-MS/MS) analyses and found that sialic acid modification was abnormal in the serum glycoprotein group of children with ASD (8). Enzymes related to sialylation are mainly divided into two categories: sialyltransferase, which catalyzes the sialic acid modification of sugar chains, and sialidase, which removes the sialic acid modification on sugar chains (9, 10). One of the recent studies reported that sialyltransferase ST3GAL5 deficient mice exhibit ASD-like behavior (11). However, the role of sialidase in ASD has not been systematically studied yet. Thus, in this study, we aim to examine the expression of sialidase in children with ASD.

There are four types of sialidases present in mammalians: neuraminidase 1 (NEU1), neuraminidase 2 (NEU2), neuraminidase 3 (NEU3), and neuraminidase 4 (NEU4) (12, 13). NEU1 is the most abundant mammalian sialidase; it primarily presents in lysosomes and acts on glycopeptides and oligosaccharides. NEU1 plays an essential role in the degradation of N-glycans (14, 15). Sialidase NEU1 deficiency has been found to affect sialic acid deposition (16–18). Moreover, NEU1 also participates in the immune system and exhibits immunomodulatory effects (19, 20).

Only a few studies focus on NEU1 expression changes in the peripheral blood in children with ASD. Therefore, we aimed to examine the NEU1 mRNA expression level in peripheral blood of children with ASD and analyze the association between NEU1 mRNA expression and ASD phenotypes to explore the relationship between NEU1 and ASD.

## Materials and Methods

### Subject Selection

We collected 42 children with ASD and 42 healthy controls with age and gender-matched. Patients were enrolled in the Child Healthcare Department of Xi ’an Children's Hospital from May to December 2019. Two experienced pediatricians made diagnoses based on DSM-5, the American Diagnostic and Statistical Manual of Mental Disorders, 5th Edition (1) and Autism Diagnostic Observation Schedule, Second Edition (ADOS-2). According to the parents' report, none of the control children had psychiatric disorders and no family history with ASD. In order to clarify whether the included children and the grouping were reasonable, a statistical analysis of the gender and age of the included children in each group was performed using statistical methods, and the results were not statistically different (Table 1). Consent forms were obtained from the parents of all participating children. This study was approved by the Ethics Committee of Xi'an Children's Hospital.

**Table 1:** Demographic and clinical variables (Means and Standard Deviations).

**Variable**	**ASD**	**Control**	***P***
Gender	37 male, 5 female	38 male, 4 female	1.0000
Age	4.050 ± 0.1039	4.083 ± 0.1708	0.8664

*P-values were calculated using the Chi-square test. ASD, autism spectrum disorder children; Control, healthy control children*.

### Blood Sampling and RT-qPCR

The classical Trizol method for total RNA extraction from peripheral blood was performed in this experiment (21). Sterility and enzyme-free consumables are guaranteed throughout the experiments. A total of 2 ml peripheral blood was collected in the EDTA anticoagulation tube from elbow veins. After transferring an appropriate amount of blood sample into a 1.5 ml Eppendorf tube, 1 ml of Trizol was added and mixed well. All blood samples were collected at the same time and under the same storage conditions. Total RNA concentration and purity measurements were performed on a Agilent BioTek Take3 Micro-Volume Plate (BioTek, Vermont, USA). Subsequently, the cDNA Reverse transcription amplification (LongGene A300 PCR, Hangzhou, China) was performed following the Goldenstar RT6 cDNA Synthesis Kit (Tsingke Biotechnology Co., Ltd, Beijing, China). The amplification procedure (BIOER FQD-96A, Hangzhou, China) was performed according to the operation of 2×T5 Fast qPCR Mix (SYBR Green I, Tsingke Biotechnology Co., Ltd, Beijing, China). The melting and amplification curves, as well as Ct values generated by the reaction, were collected, recorded, and statistically analyzed. Triplicates were performed for each sample, and the relative changes in gene expression were quantified using 2-ΔΔCt values. Primer sequences for sialidase NEU1 and the internal reference gene β-actin were designed in NCBI prime BLAST by intron spanning (Table 2) and synthesized by Tsingke Biotechnology Co., Ltd (China) with a primer concentration of 10 μM.

**Table 2:** DNA Sequences of primers used for qPCR.

**Genes primer**	**primer sequence**
NEU1 F	5′GCACATCCAGAGTTCCGAGT3′
NEU1 R	5′CAGGGTTGCCAGGGATGAAT3′
β-actin F	5′CCTTCCTGGGCATGGAGTC3′
β-actin R	5′TGATCTTCATTGTGCTGGGTG3′

*Primer sequences of sialidase (neuraminidase 1; NEU1) and the internal reference gene β-actin were designed in NCBI prime BLAST. F, forward primer; R, reverse primer*.

### ADOS-2 Evaluation

Autism Diagnostic Observation Schedule, Second Edition is a semi-structured, standardized assessment of communication, social interaction, play/ imaginative use of materials, and restricted and repetitive behaviors for individuals who have been referred because of possible ASD. The ADOS-2 has been referred to as the “gold standard” observational assessment for diagnosing ASD. ADOS-2 contains five assessment modules, which are relevant to the diagnosis of ASD at different developmental levels and chronological ages. Each module contains five domains, which are A (Language and Communication), B (Reciprocal Social Interaction), C (Play), D (Stereotyped Behaviors and Restricted Interests), and E (Other Abnormal Behaviors). Sub-entries include diagnostic items and observation items. The SA domain includes items pertaining to “Communications” and “Reciprocal Social Interactions”. The RRB domain includes items pertaining to “Restricted and Repetitive Behaviors” (22). A total of four modules of the ADOS-2 (T, 1, 2, and 3 modules) were used in this study, and the appropriate module was selected according to the age and language level of the individual. Children with ASD were evaluated by two certified developmental pediatricians using ADOS-2. Each assessment was conducted under the supervision of the child's parent or guardian. The correlation between NEU1 mRNA and ADOS-2 total diagnostic score, as well as SA and RRB score in children with ASD, were statistically analyzed.

### Statistical Analysis

To analyze the obtained data, IBM SPSS Statistics 20 was applied. A test for normality and removal of discrete values is required for each set of data from the raw results. NEU1 mRNA values that did not conform to normal distribution were expressed as the median M (range), and the nonparametric Mann-Whitney test was used for NEU1 mRNA comparison in peripheral blood of healthy controls and children with ASD. The area under the ROC curve was calculated. Pearson correlation analysis was performed to determine whether there was a correlation between NEU1 mRNA and ADOS-2 in children with ASD.

## Results

### Gene Expression Result and Receiver Operating Characteristic Analysis

**Figure 1:**
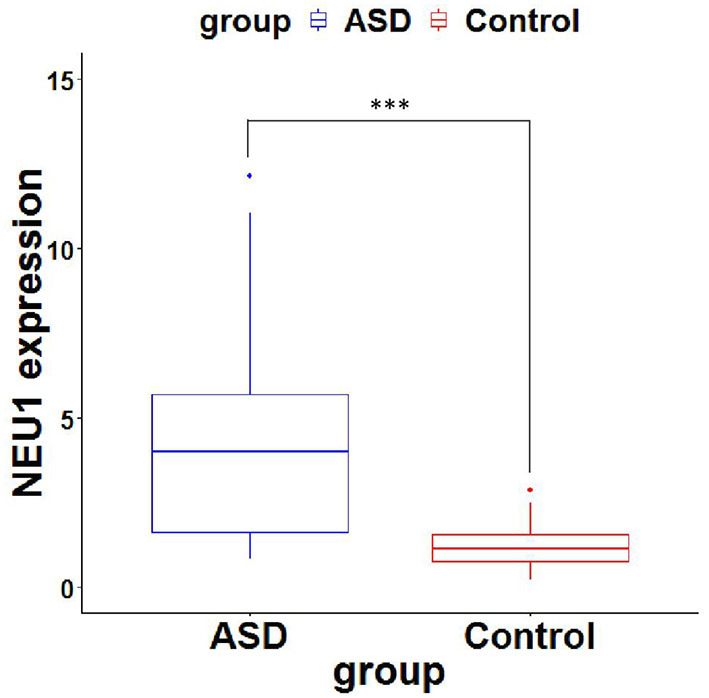
NEU1 mRNA expression (2^−ΔΔ^Ct values) of ASD and Control. *P*-values were calculated using the Mann-Whitney U test. ^***^, significant difference between ASD group and control group.

**Figure 2:**
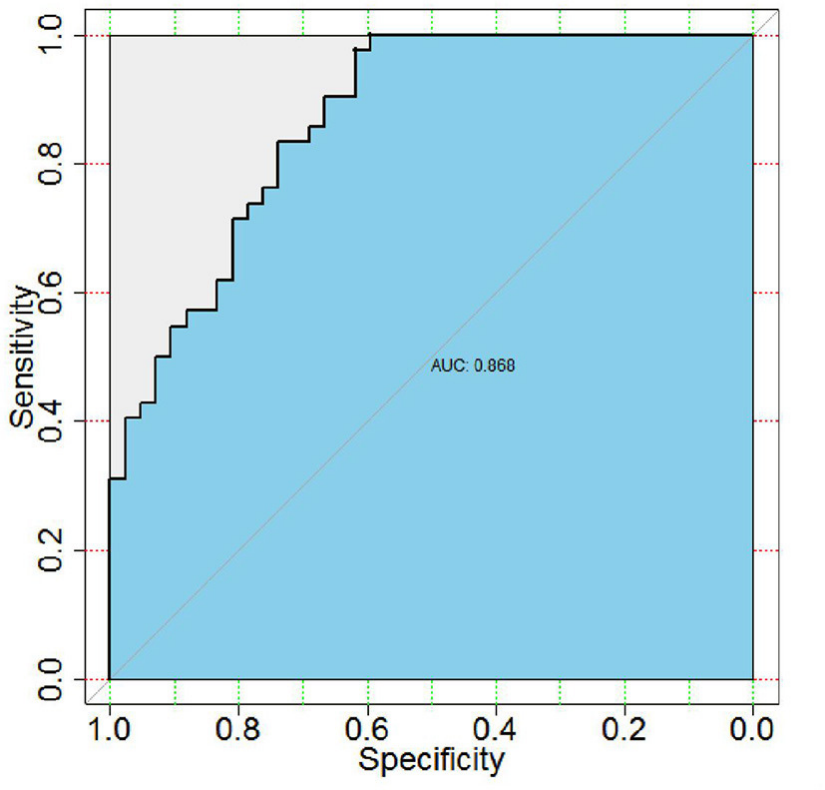
Receiver operating characteristic (ROC) curve between clinical sensitivity and specificity for every possible cut-off. ROC curves of ASD and control with NEU1 expression. AUC was 0.868 (*P* < 0.0001), sensitivity was 73.81% and specificity was 83.33%.

Neuraminidase 1 mRNA expression in peripheral blood in the ASD group [4.19 (1.588–5.767)] was significantly higher than in the Control group [1.198 (0.745–1.597)] (*P* < 0.0001) (Table 3 and Figure 1). We then calculated the ROC curve of NEU1 for all ASD samples and control samples to assess the predictive power of NEU1 levels in differentiating children with ASD from healthy controls. The analysis showed an AUC of.868 (*P* < 0.0001) with high sensitivity (73.81%) and specificity (83.33%). These results indicated the feasibility of NEU1 as a potential clinical diagnostic indicator for ASD (Figure 2).

**Table 3:** NEU1 mRNA expression between groups (*p*-values) [M (Q1~Q3)].

**Gene**	**ASD**	**Control**	***P***
NEU1	4.19 (1.588–5.767)	1.198 (0.745–1.597)	<0.0001

Statistical analysis of NEU1 expression between groups. P-values were calculated using the Mann-Whitney U test. ^***^, P <0.001 vs control.

### Correlation of ASD Phenotype With NEU1 mRNA

**Figure 3:**
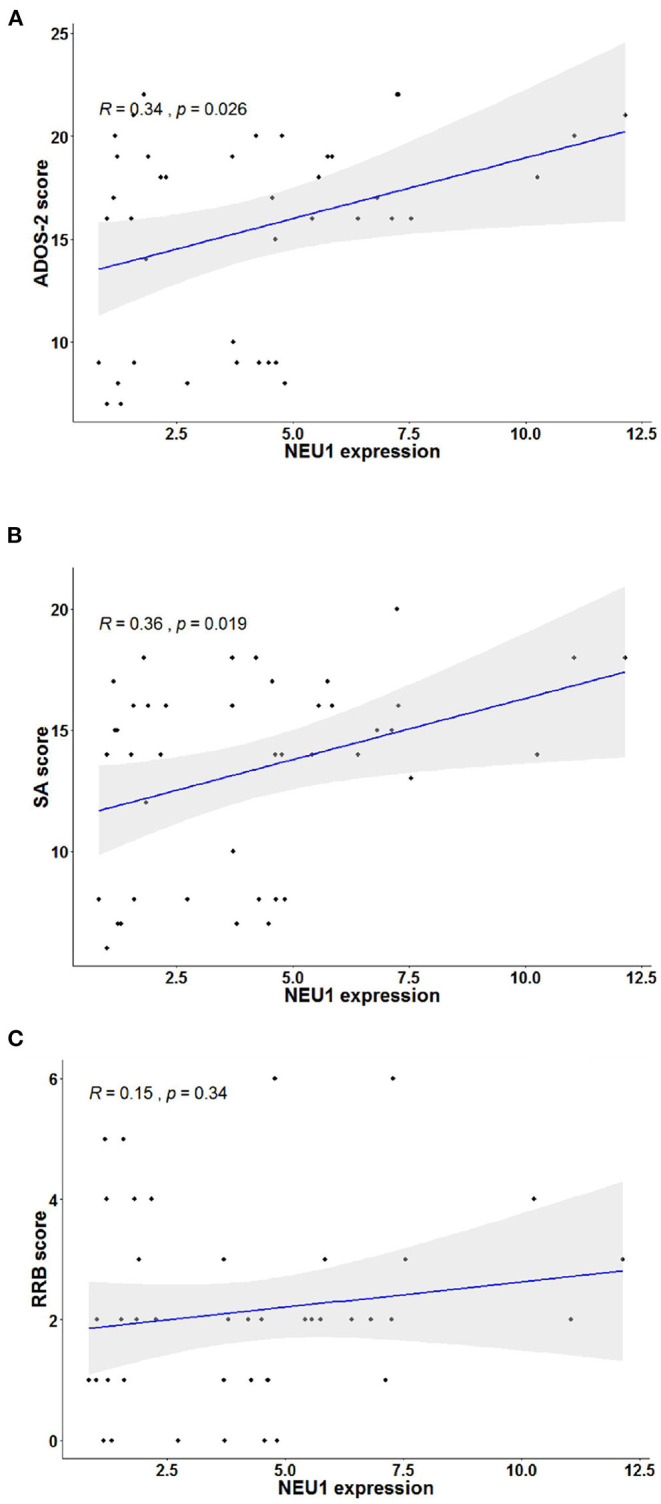
Correlation analysis in children with ASD's blood NEU1 expression with ADOS-2, SA, and RRB scores. Statistical calculation of correlation was performed on each pair to obtain the Pearson correlation result, and the sig two-tailed probability *P*-value < 0.05 represented correlation. **(A)** Correlation of NEU1 gene expression results with ADOS-2 score. **(B)** Correlation of NEU1 gene expression results with SA score. **(C)** Correlation of NEU1 gene expression results with RRB score. **(A,B)** show that the elevated expression of NEU1 was positively correlated with ADOS-2 and SA. **(C)** shows that no correlation was found with the RRB score.

To investigate the relationship between NEU1 mRNA and symptoms in children with ASD, we performed and recorded ADOS-2 scores in 42 children with ASD. The diagnostic scores of ADOS-2 and NEU1 mRNA expression levels were analyzed by Pearson correlation. We found that the ADOS-2 diagnostic score in children with ASD was correlated with the expression of NEU1 mRNA (*R* = 0.344, *P* = 0.0257) (Figure 3A). Furthermore, in order to further examine the correlation between the increased expression of NEU1 and the behavioral performance in children with ASD, we calculated the correlation of NEU1 mRNA expression level with SA and RRB in ADOS-2. The results indicated that increased expression of NEU1 mRNA was positively correlated with SA (*R* = 0.3598, *P* = 0.0193) (Figure 3B), but not with RRB (*R* = 0.15, *P* = 0.3432) (Figure 3C). The correlation of NEU1 gene expression results with ADOS-2, SA, and RRB data is listed in Table 4.

**Table 4:** Correlation of NEU1 gene expression results to Autism Diagnostic Observation Schedule, Second Edition (ADOS-2), Social Affect (SA), and Restricted and Repetitive Behavior (RRB) in children with ASD.

**Gene expression**	**ADOS-2 score**	**SA score**	**RRB score**
NEU1	*r =* 0.344 *P =* 0.0257	*r =* 0.3598 *P =* 0.0193	*r =* 0.15 *P =* 0.3432

*Statistical calculation of correlation was performed on each pair to obtain the Pearson correlation result, and the sig two-tailed probability P-value <0.05 represented correlation*.

## Discussion

In this study, we showed that the expression of NEU1 mRNA in peripheral blood was significantly increased in children with ASD compared to healthy controls. Furthermore, we demonstrated that NEU1 mRNA expression in peripheral blood could effectively distinguish children with ASD from healthy controls. In addition, we showed that the increased expression of NEU1 mRNA was positively correlated with both ADOS-2 total diagnostic score and SA score, but not with RRB score, suggesting that NEU1 alteration may be associated with ASD behavioral phenotypes, especially in social interaction deficits. Our results suggest that NEU1 may play an important role in ASD disease and lay the foundation for further studies on the relationship between NEU1 and ASD.

Our previous study focused on Maackia amurensis lectin-II (MAL-II) to study the serum proteome and serum glycoproteome in children with ASD. We found that the glycoprotein sialic acid modification in the serum of children with ASD was increased (8). In this study, our data suggested that sialidase NEU1 mRNA expression is increased in children with ASD, we speculate that the underlying mechanism is a feedback regulation on the increase of protein sialylation level caused by increased glycoprotein sialic acid modification, thereby upregulating sialidase NEU1 mRNA expression. In order to test our hypothesis, we plan to generate a mouse model to increase NEU1 expression. We will measure the protein sialylation level as well as check for ASD-related behaviors in the future. Here, we reported that NEU1, one of the sialidases responsible for removing sialic acid modifications on sugar chains, was highly expressed in ASD. One recent study also showed that the expression of plasma sialic acid is significantly reduced in ASD (23), which may affect the function of sialidase. Together, all of these recent findings support the idea that abnormal modification of glycoprotein sialylation may be one of the essential factors in the occurrence of ASD.

It has been shown that immune dysregulation is closely associated with ASD (19, 24). Sialylation plays an important role in the immune system as well (25). One recent study showed that sialyltransferase ST3GAL5 deficient mice exhibit ASD-like behaviors and dysregulated inflammatory responses. Moreover, NEU1 has been shown to functionally remove the sialic acid on TLR4, so that it will not be recognized for degradation, thereby increasing the immune response (26–28). Furthermore, sialic acid modification of TLR4 can regulate the nuclear factor kappa-B (NF-κB) signaling pathway and lead to altered the protein expression level of cytokines (29, 30). Significantly increased serum NF-κB concentration was found in children with ASD (31). Animal experiments have shown that TLR4 expression is increased in mice with maternal LPS exposure, and their offspring exhibit ASD-like behaviors (32). This may be related to the activation of microglia by TLR4 stimulation, which eventually leads to neuroinflammatory damage and neuronal death (33). Moreover, the neuroimmune response has been shown to cause ASD-like behaviors (30, 34). Overall, these results suggest that abnormal NEU1 expression may affect ASD behavior by regulating immune responses.

In addition, we tested the performance of NEU1 as a potential clinical diagnostic marker for ASD. The result of the ROC curve suggested that NEU1 mRNA increased expression in peripheral blood exhibited high sensitivity (73.81%) and specificity (83.33%) for ASD, and the area under the ROC curve was 0.868 (*P* < 0.0001). We found that the amount of NEU1 mRNA increased expression positively correlated with the severity of ASD symptoms in the diagnosed children. Studies in zebrafish reported that NEU1-KO zebrafish developed behavioral traits opposite to ASD, including excessive exploratory/boldness behavior on various tests. Furthermore, anxiety induction upregulated NEU1 expression in zebrafish (35), which is consistent with our findings.

## Limitations and Future Directions

This study found that the increase of NEU1 mRNA expression was statistically significant with SA (*P* < 0.05), but the correlation was not strong (*R* = 0.3598). Therefore, we need to further expand the sample size in the future. Meanwhile, in order to explore whether the abnormal expression of NEU1 is specific to ASD, related studies can be carried out on other common neurodevelopmental disorders in children. In addition, future research on NEU1 can explore its crucial role and related signaling pathways involved in ASD from different cells and animal models.

## Conclusion

We showed that NEU1 mRNA expression is significantly increased in children with ASD. And there is a correlation between the increased expression of NEU1 and social dysfunction in children with ASD.

## Data Availability Statement

The raw data supporting the conclusions of this article will be made available by the authors, without undue reservation.

## Ethics Statement

The studies involving human participants were reviewed and approved by Ethics Committee of Xi'an Children's Hospital. Written informed consent to participate in this study was provided by the participants' legal guardian/next of kin.

## Author Contributions

YC, LH, YY, and FK designed the project. HZ, YG, LH, HW, and DW to conduct experiments and collect data. FK, HZ, WH, and YZ for the ASD entry evaluation. YC, HZ, and YY analyzed the data and interpreted the results and wrote the manuscript. All authors participated in the revision of the manuscript, and all read and approved the submitted version. All authors provide approval for publication of the content and agree to be accountable for all aspects of the work in ensuring that questions related to the accuracy or integrity of any part of the work are appropriately investigated and resolved.

## Funding

This study was jointly supported by a grant from Shaanxi Province Key R&D Program (2020GXLH-Y-013), TCM combination with Modern Medicine of prevention and treatment of developmental brain disorders innovation team, Shaanxi University of Chinese Medicine (2019-YL07), National Natural Science Foundation of China (NSFC 81371900), The Shaanxi Provincial Science and Technology Research and Development Program (213ST2-09), Shaanxi Province Key R&D Program (2021SF-194), Xi'an Science and Technology Plan Project [20YXYJ0006(4)], and Natural Science Basic Research Program of Shaanxi (2022JQ-979).

## Conflict of Interest

FK was employed by LIH Healthcare. The remaining authors declare that the research was conducted in the absence of any commercial or financial relationships that could be construed as a potential conflict of interest.

## Publisher's Note

All claims expressed in this article are solely those of the authors and do not necessarily represent those of their affiliated organizations, or those of the publisher, the editors and the reviewers. Any product that may be evaluated in this article, or claim that may be made by its manufacturer, is not guaranteed or endorsed by the publisher.
