# Supplementary material for: Correlation Between Sialidase NEU1 mRNA Expression Changes in Autism Spectrum Disorder
Source: Front Psychiatry. 2022 Jun 9;13:870374. doi: 10.3389/fpsyt.2022.870374 (PMC9218098; doi:10.3389/fpsyt.2022.870374)
Supplement: Supplementary file 1 [file Table_1.DOCX]

**Supplementary Material:**

Since ASD is more prevalent in males than in females it would be interesting to include an additional statistic containing only the data of male children. The results of male children were consistent with the results in entire group. The supplementary results are shown in the supplementary Tables 1-3.

Supplementary Table 1

Demographic and clinical variables (Means and Standard Deviations).

| VARIABLE | ASD | Control | P |
| --- | --- | --- | --- |
| Gender | 37male | 38male | 1.0000 |
| Age | 4.07±0.7119 | 4.07±1.078 | 0.9987 |

Supplementary Table 2

Gene expression p value for NEU1 types between groups[M (Q1~Q3)]

| Gene | ASD | Control | P |
| --- | --- | --- | --- |
| NEU1 | 4.377 (1.745~5.981) | 1.201 (0.8892~1.69) | < 0.0001 |

Supplementary Table 3

Correlation of NEU1 gene expression results with ADOS-2、SA and RRB boys in ASD patients.

| Gene expression | ADOS-2 score | SA score | RRB score |
| --- | --- | --- | --- |
| NEU1 | r=0.348  P=0.0323 | r=0.3693  P=0.0225 | r=0.1352  P=0.4182 |

Amplification and melting curves

The amplification curve is a dynamic process of the qPCR reaction, and the melting curve should show a single peak and appear above 80 °C, indicating that the specificity of the amplification product is good. The amplification curve and dissolution curve of neu1 and the reference gene β-actin are shown in the following figures (supplementary material Figures 1-4).


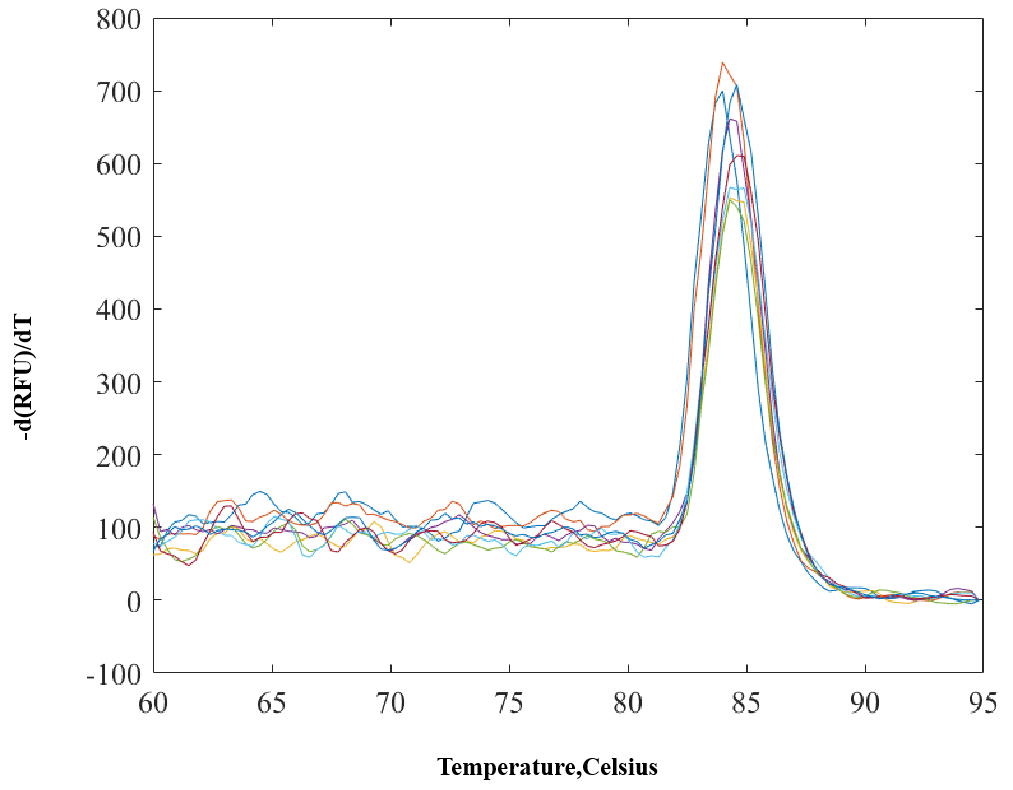


Supplementary Material Figure 1 Melting curves of NEU1


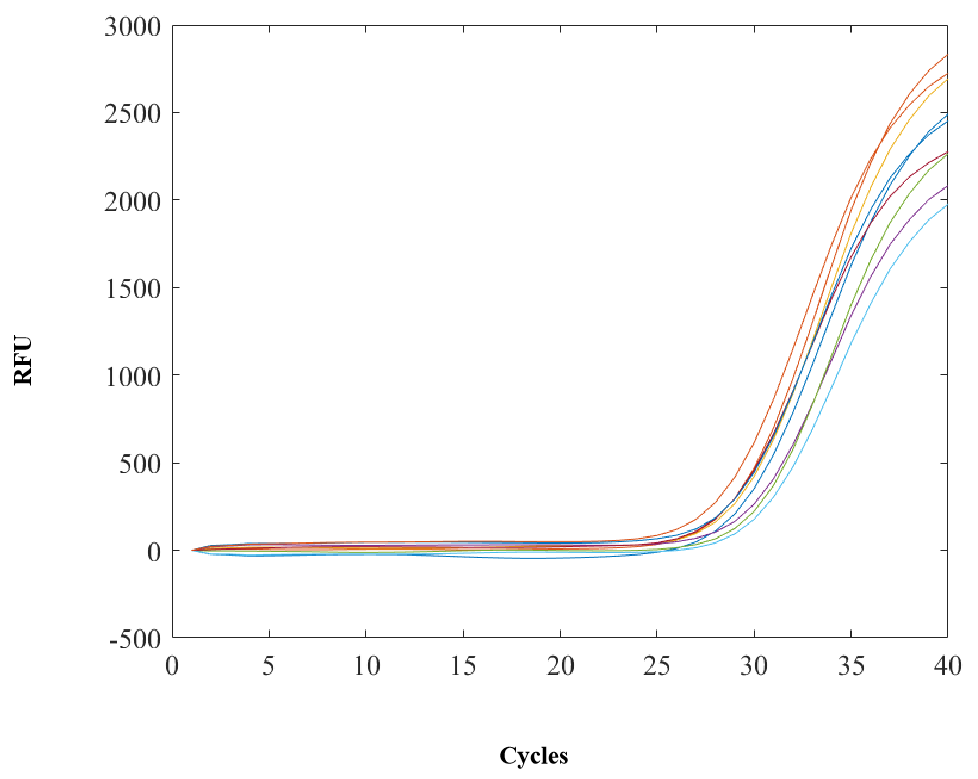


Supplementary Material Figure 2 Amplification curves of NEU1


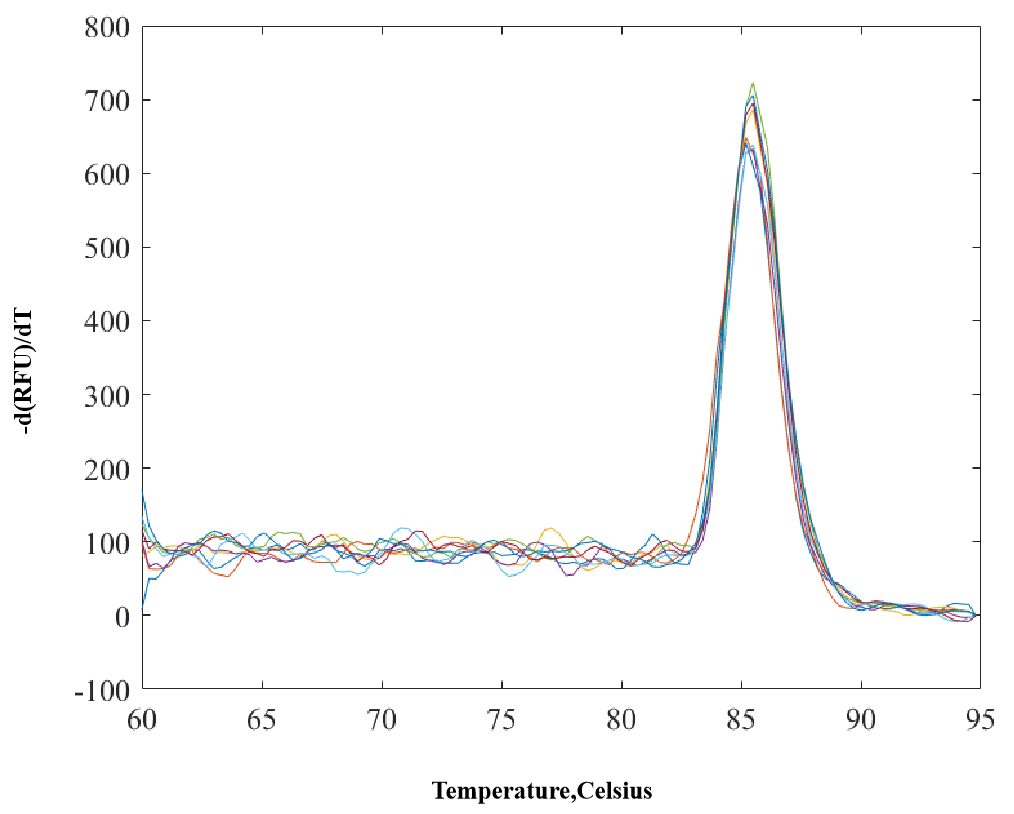


Supplementary Material Figure 3 Melting curves of β-actin


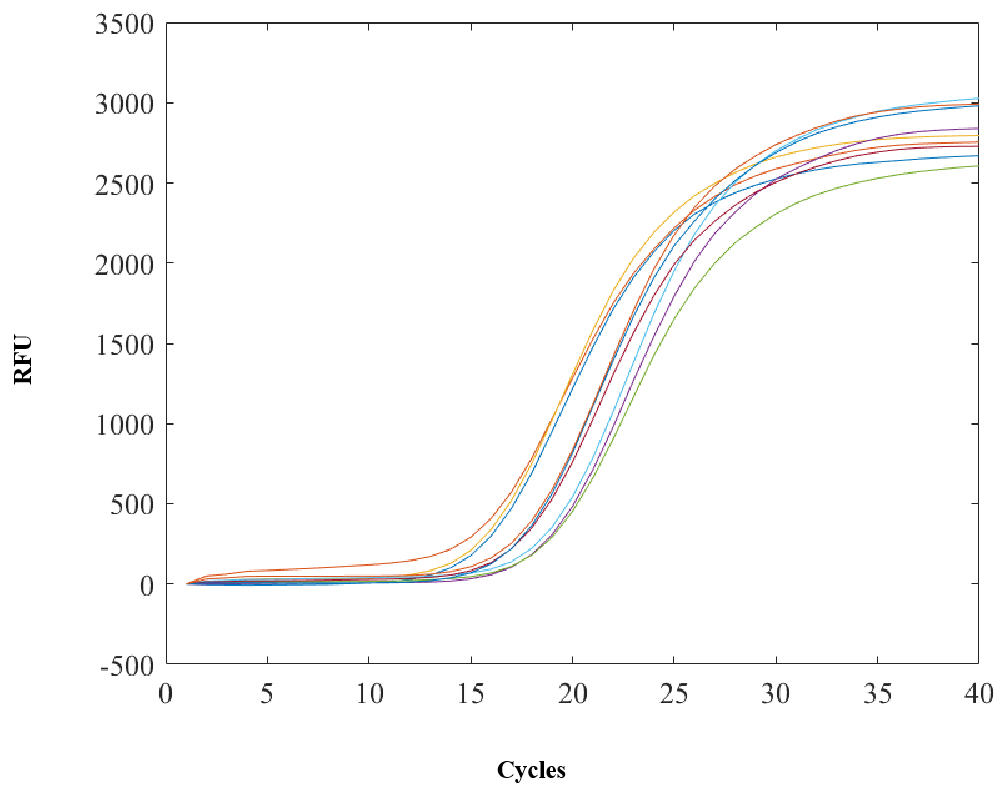


Supplementary Material Figure 4 Amplification curves of β-actin
